# The Reliability of the Use of Serum Neutrophil Gelatinase–Associated Lipocalin Levels in the Assessment of Renal Functions after Coronary Artery Bypass Grafting

**DOI:** 10.1155/2018/7291254

**Published:** 2018-03-05

**Authors:** Ozan Onur Balkanay, Deniz Göksedef, Suat Nail Ömeroğlu, Gökhan İpek

**Affiliations:** Cerrahpasa Medical Faculty, Department of Cardiovascular Surgery, Istanbul University, Istanbul, Turkey

## Abstract

**Objective:**

Evaluation of perioperative renal function is very important for early diagnosis and treatment of acute kidney injury after coronary artery bypass grafting. Serum creatinine levels, creatinine clearance, and estimated glomerular filtration rates used in determination of postoperative kidney injury can lead to late detection. Therefore, it is necessary to make a diagnosis earlier in clinical practice and to search for a reliable method. The reliability of the use of serum neutrophil gelatinase–associated lipocalin levels in close follow-up of renal function was evaluated in patients with coronary artery bypass grafting under cardiopulmonary bypass in our study.

**Patients and Methods:**

A total of 40 patients who underwent coronary artery bypass grafting under cardiopulmonary bypass between September 2009 and February 2010 were included in the study. The reliability of the postoperative 1st day plasma neutrophil gelatinase–associated lipocalin (Triage^®^ NGAL Device; Biosite Inc.) measurements was evaluated in kidney injury developed in the first 5 days after operation that was detected using the Risk-Injury-Failure-Loss-End stage criteria.

**Results:**

Ten (25%) women and 30 (75%) male patients were included in the study. The average age is 59 ± 8.6 years. Kidney injury according to Risk-Injury-Failure-Loss-End stage criteria developed in 8 patients (20%). For 150 ng/mL cutoff value of postoperative plasma neutrophil gelatinase–associated lipocalin levels, the area under the receiver-operating characteristic curve was 0.965. Neutrophil gelatinase–associated lipocalin's sensitivity, specificity, and negative and positive predictive values were 100%, 93.8%, 100%, and 80%, respectively.

**Conclusion:**

It has been determined that plasma neutrophil gelatinase–associated lipocalin levels can be reliably used for early diagnosis of kidney dysfunction in patients undergoing coronary artery bypass grafting.

## 1. Introduction

One of the leading complications following cardiac surgery is acute kidney injury [1]. Cardiopulmonary bypass (CPB) is the main factor in this complication. In multicenter studies, cardiac surgery performed under CPB is shown as the second most common cause of acute kidney injury (AKI) after nephrotoxic drug use [2, 3]. AKI after cardiac surgery is known to increase postoperative mortality and morbidity [4–7]. The main reason for this poor prognosis is that early detection is not possible and effective early treatment is not performed [8, 9]. Serum creatinine level measurements for long years have been used as the main indicator of kidney dysfunction [5]. However, the use of serum creatinine levels in the early detection of acute kidney injury is less sensitive [5]. Serum creatinine levels can be normal even if there is over 50% damage in the kidneys [5]. There is a study showing that 41.1% of the cases can be underdiagnosed by means of AKI according to the serum creatinine level [8]. Additionally, the reliability of the estimated glomerular filtration rate (GFR) measurements to indicate the creatinine clearance rate in patients undergoing coronary artery bypass grafting (CABG) is suboptimal [10]. For these reasons, new researches have been conducted and new biomarkers, which can detect early kidney damage and enable early intervention, continued to be investigated. Recent studies indicate that measurement of the neutrophil gelatinase–associated lipocalin (NGAL) level is one of the early biomarkers that can be used to detect CPB-associated AKI [5, 11]. The aim of our study was to investigate the reliability of the use of serum NGAL levels in the early detection of AKI after CABG under CPB.

## 2. Patients and Methods

A total of 40 consecutive patients without preexisting renal failure undergoing CABG under CPB between September 2009 and February 2010 were included in the study, who did not have exclusion criteria and were operated by the same surgical team. The kidney injury during the 5-day follow-up period was determined according to the Risk-Injury-Failure-Loss-End stage (RIFLE) criteria, creatinine clearance rate measurements, and estimated glomerular filtration rate measurements according to the Cockcroft–Gault equation, and the reliability of the postoperative first day plasma NGAL values for renal dysfunction was retrospectively investigated [12].

In the RIFLE criteria, there are 5 subcategories of kidney dysfunction: *risk* (1.5-fold increased serum creatinine levels or GFR decrease higher than 25% or urine output lower than 0.5 mL/kg/h during 6 hours), *injury* (2-fold increased serum creatinine levels or GFR decrease higher than 50% or urine output lower than 0.5 mL/kg/h during 12 hours), *failure* (3-fold increased creatinine levels higher than or equal to 4 mg/dL or acute rise higher than or equal to 0.5 mg/dL or GFR decrease of 75% or urine output lower than 0.3 mL/kg/h during 24 hours or anuria during 12 hours), *loss* (persistent acute renal failure and complete loss of renal function more than 4 weeks), and *ESRD* (end-stage renal disease more than 3 months) [12].

Concomitant surgical procedures, off-pump surgery, presence of the history of antithrombotic agent usage during 2 weeks preoperatively, presence of accompanying acute infection, presence of chronic infection, presence of preoperative advanced liver or pulmonary insufficiency, and preexisting renal failure were considered among the exclusion criteria.

### 2.1. Acute Kidney Injury

The definition of AKI is used as a general definition for acute kidney dysfunction [5]. Acute Dialysis Quality Initiative identifies AKI within the RIFLE criteria [12, 13]. In our study, kidney injury was detected according to the RIFLE criteria [12]. The RIFLE classification includes both glomerular filtration rate (GFR) and urine output (UO) criteria. Injury category is defined as a 2-fold increase in serum creatinine level or GFR decrease higher than 50% or UO lower than 5 mL/kg/h during 12 hours.

### 2.2. Plasma Neutrophil Gelatinase–Associated Lipocalin Measurement

The NGAL measurements were performed at the time of intensive care follow-up at 24 hours after termination of CPB using the Triage NGAL Test Device (Biosite Inc., San Diego, CA, USA). The cutoff value was accepted as 150 ng/mL in the measurements.

### 2.3. Statistical Analysis

Categorical variables were expressed as frequency and percentage, and continuous variables were expressed as mean ± standard deviation (95% confidence interval). *P* value of less than 0.05 was considered significant. IBM SPSS software package version 21.0 (SPSS, Chicago, IL, USA) was used for statistical analysis. The receiver-operating characteristic (ROC) curve was drawn to calculate the area under the curve (AUC). Values above 0.7 for AUC-ROC were considered to have good test performance. Sensitivity, specificity, and positive and negative predictive values of serum NGAL measurements for detecting AKI were calculated.

### 2.4. Primary End Point

The calculation of the confidence of the serum NGAL measurement based on the 150 ng/mL cutoff value of the blood sample taken at 24 hours following termination of the CPB in detecting AKI according to the RIFLE criteria was accepted as the primary end point of the study.

## 3. Results

Ten (25%) women and 30 (75%) male patients were included in the study. The average age was 58.7 ± 8.6 (56–61.2) years (Table 1). The mean body mass index of the patients was 28.5 ± 4.3 (27.3–30) kg/m^2^, and the mean body surface area was 1.9 ± 0.2 (1.8–1.9) m^2^ (Table 1). A total of 35 patients (87.5%) had hypertension, 26 patients (65%) had hyperlipidemia, and 16 patients (40%) had diabetes mellitus. All patients had CABG indication and underwent elective on-pump CABG surgery. None of them had a preoperative kidney dysfunction history.

**Figure 1:**
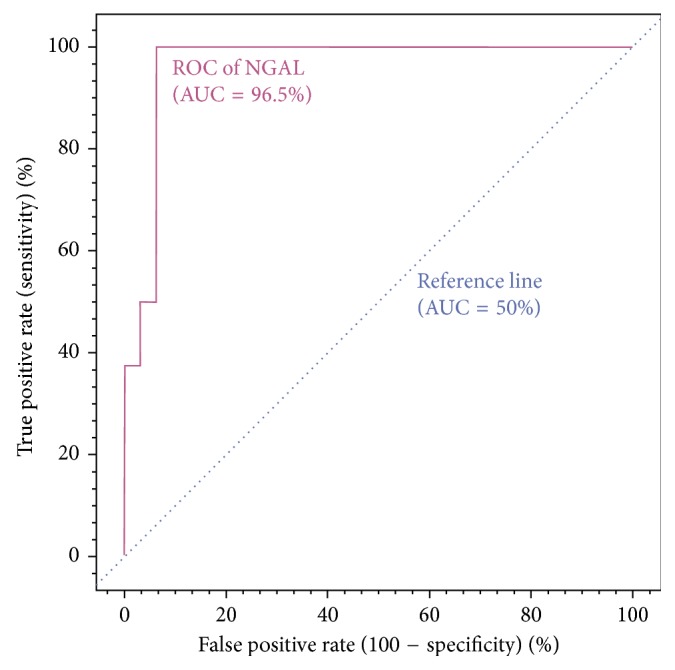
ROC curve of NGAL levels (cutoff value = 150 ng/mL) on postoperative day 1 for diagnosis of kidney injury according to the RIFLE criteria. AUC: area under the curve; NGAL: neutrophil gelatinase–associated lipocalin; RIFLE: Risk-Injury-Failure-Loss-End stage; ROC: receiver-operating curve.

The mean ± standard deviation (95% CI) values of creatinine clearance rates on preoperative and postoperative 1st and 5th days were 85.7 ± 30 (76.9–94.5), 109.4 ± 42 (97.8–121.8), and 87.8 ± 32.8 (77.7–97.7) mL·min^−1^·1.73 m^2^, respectively (Table 2). Kidney injury according to the RIFLE criteria was developed in 8 patients (20%) during the postoperative 5-day period. At the 150 ng/mL cutoff value of the postoperative first day plasma NGAL level, the AUC-ROC was 0.965 (Figure 1). The sensitivity, specificity, and negative and positive predictive values of NGAL on the postoperative first day in predicting the kidney injury in the period of postoperative 5 days were found as 100%, 93.8%, 100%, and 80%, respectively.

## 4. Discussion

There is an intense relationship between on-pump CABG and the development of the postoperative kidney injury. It is stated that AKI can be developed at high levels up to 30–50% after open-heart surgery under CPB [5, 14, 15]. However, kidney failure requiring dialysis is observed in approximately 1-2% of patients after cardiac surgery, and this is associated with about 60% mortality [16, 17]. The leading risk factors contributed to develop AKI after open-heart surgery under CPB are preoperative kidney dysfunction, advanced age, hypertension, diabetes mellitus, impaired left ventricular ejection fraction, perioperative hemodynamic instability, long-term CPB support, vasoconstriction, development of reperfusion injury, reduction of renal blood flow, presence of nonpulsatile flow, embolization of atherosclerotic plaque, perioperative hypothermia and perfusion-dependent cascade activation with extensive inflammatory response, and postoperative intra-aortic balloon pump usage [5, 16, 18]. Serum creatinine and estimated glomerular filtration rate calculations are generally used as comparison parameters in various retrospective studies in the current literature [5]. However, 24-hour creatinine clearance rate measurements as the basic comparative parameter could be more reliable. Besides, the reliability of estimated glomerular filtration rate calculations in predicting the actual creatinine clearance rate is not so high [10]. Therefore, we used 24-hour creatinine clearance rate measurements in the early postoperative period as the gold standard in addition to urinary output and creatinine criteria of the RIFLE as comparison parameters. Measurements such as serum creatinine, estimated glomerular filtration rate, or creatinine clearance rate (which requires 24-hour urine accumulation) reflect the onset of AKI late and require the development of biomarkers that detect acute kidney damage earlier [5]. Additionally, this requirement is more available for patients undergoing cardiac surgery under CPB where AKI is frequently encountered [19]. NGAL was identified as the biomarker with the highest positive predictive value alone in a study performed with many new biomarkers [20]. NGAL is a protein and a member of lipocalin family with a molecular weight of 25 kDa [1, 5]. It is secreted from neutrophils and organ epithelium such as the kidney, uterus, prostate, and trachea [21]. Epithelial damage increases the serum and urinary NGAL levels. Infection, sepsis, and active inflammatory processes can also elevate the levels of NGAL. However, NGAL is less sensitive to infection and therefore not used in clinical practice in terms of infection [22, 23]. NGAL is rarely detected in plasma and urine in situations of healthy kidneys [5]. However, in cases of acute tubular injury, NGAL is detected both in serum and in urine and increases markedly [24]. NGAL is elevated as detectable in both serum and urine at 2 hours postischemically, and the elevation level correlates with the duration of renal ischemia [25]. NGAL reaches peak levels in both plasma and urine at 2–6 postoperative hours. Previous studies have shown that the AUC-ROC of the postoperative 2nd hour urinary NGAL measurement is between 0.85 and 0.871 in detecting the development of AKI after cardiac surgery [1, 20]. Besides, the AUC-ROC of the plasma NGAL level for detecting AKI is 0.64. However, in this study, it appears that blood plasma samples are taken immediately after the termination of the CPB [26]. It should be noted that the levels of NGAL for blood serum and urine measurements reach peak levels within 2–4 hours. Therefore, the peak timing for NGAL measurements after cardiac surgery was usually at the time the patient was admitted to the intensive care unit [1, 9]. It was predicted that NGAL may detected the CPB-related kidney dysfunction during the early intensive care unit period. As a result of a meta-analysis, it was stated that the AUC-ROC of plasma NGAL measurements for the detection of AKI was 0.71 for all studies and 0.73 for studies based on the RIFLE criteria [27]. In the previous studies performed, the sensitivity and specificity of plasma NGAL at postoperative 3 hours were 94.1% and 93.9%, respectively [28]. By the help of the use of biomarkers that can detect AKI early, kidney damage can be reversed with effective interventions [8]. At this point, early detection of subclinical tubular injury is important [29]. NGAL can be used in the early detection of CPB-related AKI, thus making it possible to implement early interventional strategies. Current literature suggests that NGAL can be used as an early biomarker in the detection of acute kidney damage after CPB [5]. In parallel with these findings, we found that measurements of the plasma NGAL level on the first postoperative day could be used as an early biomarker in the detection of AKI after CPB with high sensitivity and specificity values in reflecting the period of postoperative five days.

## 5. Conclusions

It has been determined that plasma NGAL levels can be reliably used postoperatively for early diagnosis of kidney dysfunction in patients undergoing on-pump CABG. However, these promising results must be confirmed with further multicenter studies with larger patient populations.

## Figures and Tables

**Table 1:** Perioperative patient variables.

Variable	*n*	%	Mean ± SD	Min.–max.	95% CI
*Preoperative*					
Age (year)			58.7 ± 8.6	40–79	56–61.2
Female gender (F)	10	25			
Body mass index (kg/m^2^)			28.5 ± 4.3	23–42	27.3–30
Body surface area (m^2^)			1.9 ± 0.2	1.5–2.3	1.8–1.9
Hypertension	35	87.5			
Hyperlipidemia	26	65			
Diabetes mellitus	16	40			
*Intraoperative*					
Aortic cross clamping time (min)			60.2 ± 28.1	12–110	51.2–68.7
Total perfusion time (min)			92.4 ± 34.9	35–151	81.8–102.9
Distal bypass			2 ± 0.9	1–4	1.8–2.3
*Postoperative*					
Extubation time (hour)			14 ± 14.4	4–95	10.7–18.9
ICU follow-up (hour)			49.5 ± 19.1	40–120	44.2–55.9
Kidney injury^∗^	8	20			
Hospital stay (day)			8.4 ± 3	6–19	7.6–9.4

CI: confidence interval; F/M: female/male; ICU: intensive care unit; Min.: minimum; Max.: maximum; SD: standard deviation; ^∗^during the postoperative 5-day period according to the RIFLE (Risk-Injury-Failure-Loss-End stage) classification.

**Table 2:** Perioperative kidney function tests.

Variable	Pre-op, mean ± SD	95% CI	PO 1st day, mean ± SD	95% CI	PO 5th day, mean ± SD	95% CI
BUN (mg/dL)	40.4 ± 13	36.2–44.2	41 ± 15.9	36–45.6	40.1 ± 18.4	35.1–46.1
Blood creatinine (mg/dL)	1.1 ± 0.2	1–1.1	1 ± 0.3	0.9–1	1 ± 0.3	0.9–1.1
Total urine output during 24 h (mL)	1909 ± 1073	1596–2258	2885 ± 822	2630–3151	2789 ± 1444	2360–3205
CCR (mL·min^−1^ per 1.73 m^2^)	85.7 ± 30	76.9–94.5	109.4 ± 42	97.8–121.8	87.8 ± 32.8	77.7–97.7
GFR (mL·min^−1^ per 1.73 m^2^)	77.5 ± 18.9	71.9–83.1	89.7 ± 27.9	80.9–98.3	81.2 ± 19.7	75.3–87.1

BUN: blood urea nitrogen; CCR: creatinine clearance rate; CI: confidence interval; GFR: estimated glomerular filtration rate measurement of the Cockcroft–Gault equation adjusted to the body surface area; Pre-op: preoperative; PO: postoperative; SD: standard deviation.

## Additional Points

*Limitations of Our Study*. The total number of patients enrolled in our study and retrospective, single-center conduction are major limitations of our study.
